# In this month’s Bulletin

**DOI:** 10.2471/BLT.18.000418

**Published:** 2018-04-01

In the editorial section, Carla AbouZahr et al. (226) introduce an upcoming series on civil registration and vital statistics. 

Tatum Anderson (229–230) reports on moves to improve cancer care provision in Botswana, Kenya and Rwanda. Fiona Fleck (231–232) interviews Randall Packard about the lessons of global health histories. 

## China

### More than 2.5 microns

Lijian Han et al. (233–242) estimate exposure to varied components of air pollution.

## Ethiopia

### Untreated depression and tuberculosis

Fentie Ambaw et al. (243–255) study impacts on treatment outcomes, disability, and quality of life. 

## Malawi

### Preventing mother-to-child-transmisssion

Monique van Lettow et al. (256–265) analyse missteps in HIV prevention programmes.

## Nepal

### Recovering from an earthquake

Sophie Goyet et al. (286–291) describe support provided to district health services.

## Global

### Improving in-hospital use of antibiotics

Christophe Van Dijck et al. (266–280) review the evidence for antibiotic stewardship interventions.

### Appreciating age

Alana Margaret Officer and Vânia de la Fuente Núñez (295–296) describe the effects of stereotypes used to describe older people.

### Regulating biosimilars

Hye-Na Kang and Ivana Knezevic (281–285) detail evaluation methods needed to ensure safe and effective products. 

### Increased sequencing data

Dennis Carroll et al. (292–294) introduce the global virome project.

